# Object similarity affects the perceptual strategy underlying invariant visual object recognition in rats

**DOI:** 10.3389/fncir.2015.00010

**Published:** 2015-03-12

**Authors:** Federica B. Rosselli, Alireza Alemi, Alessio Ansuini, Davide Zoccolan

**Affiliations:** ^1^Visual Neuroscience Lab, International School for Advanced Studies (SISSA)Trieste, Italy; ^2^Department of Applied Science and Technology, Center for Computational Sciences, Politecnico di TorinoTorino, Italy; ^3^Human Genetics FoundationTorino, Italy

**Keywords:** object recognition, rodent vision, invariance, perceptual strategy, view-invariant, view-dependent

## Abstract

In recent years, a number of studies have explored the possible use of rats as models of high-level visual functions. One central question at the root of such an investigation is to understand whether rat object vision relies on the processing of visual shape features or, rather, on lower-order image properties (e.g., overall brightness). In a recent study, we have shown that rats are capable of extracting multiple features of an object that are diagnostic of its identity, at least when those features are, structure-wise, distinct enough to be parsed by the rat visual system. In the present study, we have assessed the impact of object structure on rat perceptual strategy. We trained rats to discriminate between two structurally similar objects, and compared their recognition strategies with those reported in our previous study. We found that, under conditions of lower stimulus discriminability, rat visual discrimination strategy becomes more view-dependent and subject-dependent. Rats were still able to recognize the target objects, in a way that was largely tolerant (i.e., invariant) to object transformation; however, the larger structural and pixel-wise similarity affected the way objects were processed. Compared to the findings of our previous study, the patterns of diagnostic features were: (i) smaller and more scattered; (ii) only partially preserved across object views; and (iii) only partially reproducible across rats. On the other hand, rats were still found to adopt a multi-featural processing strategy and to make use of part of the optimal discriminatory information afforded by the two objects. Our findings suggest that, as in humans, rat invariant recognition can flexibly rely on either view-invariant representations of distinctive object features or view-specific object representations, acquired through learning.

## Introduction

Over the past few years, rat vision has become the subject of intensive investigation (Zoccolan et al., 2009, 2010; Meier et al., 2011; Tafazoli et al., 2012; Vermaercke and Op de Beeck, 2012; Alemi-Neissi et al., 2013; Brooks et al., 2013; Meier and Reinagel, 2013; Reinagel, 2013a,b; Wallace et al., 2013; Vermaercke et al., 2014; Vinken et al., 2014), because of the experimental advantages that rodent species might offer as models to study visual functions (see Zoccolan, 2015 for a review). Recent studies have found that rats are capable of invariant (a.k.a. transformation-tolerant) recognition, i.e., they can recognize visual objects in spite of substantial variation in their appearance (Zoccolan et al., 2009). This ability has been found to rely on the spontaneously perceived similarity between novel and previously learned views of an object, as well as on the gradual, explicit learning of each newly encountered view (Tafazoli et al., 2012). This suggests that rats achieve invariant object recognition by combining the automatic tolerance afforded by partially invariant representations of distinctive object features with the more complete invariance acquired by learning and storing multiple, view-specific object representations.

This account is in agreement with the large body of experimental and theoretical work on human visual object recognition. Following a decade of debate about whether human object vision is better accounted for by view-invariant (structural description) or view-based theories (Biederman and Gerhardstein, 1995; Tarr and Bülthoff, 1995; Hayward and Tarr, 1997; Hayward, 2003), most investigators now agree that view-invariant feature detectors and view-specific object representations can be both employed by the visual system (under different circumstances) to achieve invariant recognition (Tarr and Bülthoff, 1998; Lawson, 1999; Hayward, 2003). In fact, it has been shown that humans display view-invariant recognition of familiar objects, but have a view-dependent performance in recognition tasks involving novel objects or unfamiliar object views (Edelman and Bülthoff, 1992; Spetch et al., 2001). Nonetheless, even novel objects or object views can be recognized in a view-invariant manner, if they contain distinctive features that remain “diagnostic” of object identity despite (e.g.) rotation in the image plane (Tarr et al., 1997; Lawson, 1999; Spetch et al., 2001; Wilson and Farah, 2003). More in general, it has been proposed that recognition ranges from view-invariant to view-dependent, depending on how demanding is the object discrimination task (Newell, 1998; Hayward and Williams, 2000; Vuong and Tarr, 2006). Several studies suggest that the same argument applies to the recognition strategies of other species, e.g., monkeys (Logothetis et al., 1994; Logothetis and Pauls, 1995; Wang et al., 2005; Nielsen et al., 2008; Yamashita et al., 2010) and pigeons (Wasserman et al., 1996; Spetch et al., 2001; Spetch and Friedman, 2003; Gibson et al., 2007), although a number of differences with human recognition (in addition to commonalities) has also been found (e.g., see Soto and Wasserman, 2014 for a review).

**Figure 1:**
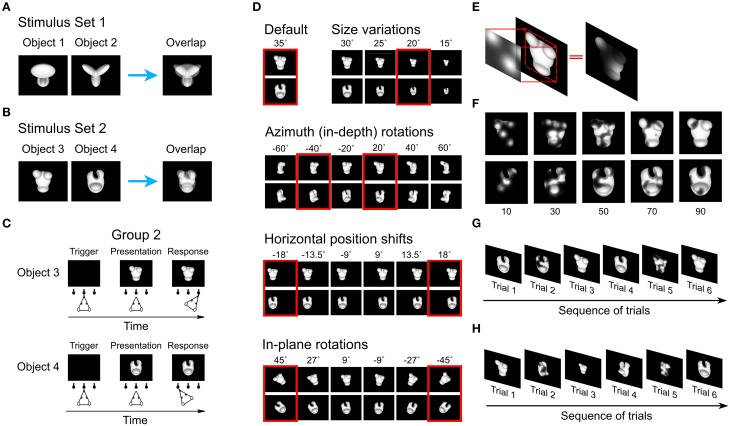
**Visual objects, behavioral task and the Bubbles method. (A)** Default views of the two objects that rats were trained to discriminate in Alemi-Neissi et al. (2013). In the present study, these objects are referred to as Object 1 and 2 and, collectively, as Stimulus Set 1. The panel on the right shows to what extent these views of the objects overlapped, when superimposed. **(B)** Default views of the two objects that rats were trained to discriminate during Phase I of the present study. These objects are referred to as Object 3 and 4 and, collectively, as Stimulus Set 2. The panel on the right shows to what extent these views of the objects overlapped, when superimposed. **(C)** Schematic of the object discrimination task. Rats were trained in an operant box that was equipped with an LCD monitor for stimulus presentation and an array of three sensors. The animals learned to trigger the presentation of a visual object by licking the central sensor, and to associate the identity of each object to a specific reward port/sensor (right port for Object 3 and left port for Object 4). **(D)** A sample of the transformed object views used during Phase II of the study. Transformations included: (1) size changes; (2) azimuth in-depth rotations; (3) horizontal position shifts; and (4) in-plane rotations. Azimuth rotated and horizontally shifted objects were also scaled down to a size of 30° of visual angle; in-plane rotated objects were scaled down to a size of 32.5° of visual angle. Note that each transformation axis was sampled more densely than shown in the figure—sizes were sampled in 2.5° steps; azimuth rotations in 5° steps; position shifts in 4.5° steps; and in-plane rotations in 9° steps. The red frames highlight the subsets of object views that were tested in bubbles trials. **(E)** Illustration of the Bubbles method, which consists in generating an opaque mask (fully black area) punctured by a number of randomly located windows (i.e., the bubbles; shown as semi-transparent, circular openings) and then overlapping the mask to the image of a visual object, so that only parts of the object is visible through the mask. **(F)** Examples of the different degrees of occlusion that can be achieved by varying the number of bubbles in the masks. **(G)** An example of possible trials' sequence at the end of experimental Phase I. The object default views were presented both unmasked and masked in randomly interleaved trials (named, respectively, regular and bubbles trials). **(H)** An example of possible trials' sequence during experimental Phase II. The animals were presented with interleaved regular and bubbles trials. The former included all possible unmasked object views to which the rats had been exposed up to that point (i.e., size changes and azimuth rotations in this example), whereas the latter included masked views of the most recently trained transformation (i.e., −40° azimuth rotated objects).

While performance-based studies (as many of those mentioned above) can assess to what extent object recognition, in a given task, is transformation-tolerant, the question of what object features are selected to recognize an object, and whether the same features are relied upon, across different object views, as preferential markers of object identity can be more directly addressed by the use of classification image methods (Nielsen et al., 2006, 2008; Vermaercke and Op de Beeck, 2012; Alemi-Neissi et al., 2013). In a recent study, we have used one of such approaches (the Bubbles method; Gosselin and Schyns, 2001) to show that the diagnostic visual features underlying rat discrimination of two multi-lobed visual objects (see Figure 1A, left panels) remained remarkably stable across a variety of transformations—translation, scaling, in-plane and in-depth rotation. This result, while consistent with a view-invariant representation of diagnostic object features, does not rule out the possibility that, under more challenging conditions (e.g., discrimination of very similar objects), rat recognition may become more view-dependent. The goal of the present study was to test this hypothesis and provide a quantitative comparison between the recognition strategies used by rats under two different levels of object discriminability.

We trained a group of rats to discriminate a new pair of multi-lobed objects (see Figure 1B, left panels), presented across a range of sizes, positions, in-depth rotations and in-plane rotations. Compared to the object pair used in our previous study (shown in Figure 1A, left panels), these new objects were more similar to one another at the pixel level and were made of less distinctive structural parts. The recognition strategies underlying discrimination of this new object pair was uncovered using the Bubbles method, and the results were compared with those reported in our previous study. New analyses of the previous set of data were also performed, so as to thoroughly quantify the influence of stimulus structure on object recognition strategy.

Our results show that, in contrast to what we observed under conditions of high stimulus discriminability, where rats relied on a largely view-invariant, multi-featural recognition strategy, discrimination of structurally similar objects led to a more view-dependent and subject-dependent, albeit still multi-featural, object processing strategy.

## Materials and methods

With the exception of the visual stimuli and some of the data analyses, the materials and methods used in this study are the same as those used in Alemi-Neissi et al. (2013). As such, we provide here a short description only and we invite the reader to refer to our previous study for a complete account.

### Subjects

Six adult male Long Evans rats (Charles River Laboratories) were tested in a visual object discrimination task. Animals were 8 weeks old at their arrival and weighted approximately 250 g. They typically grew to over 600 g over the course of the study. Rats had free access to food but were water-deprived during the days they underwent behavioral training, that is, they were dispensed with 1 h of water *pro die* after each experimental session, and received an amount of 4–8 ml of pear juice as reward during the training. Note that, out of these six rats, only three reached the criterion performance to be admitted to the main experimental phases (i.e., 70% correct discrimination of the default views of the target objects shown in Figure 1B). Therefore, only three out of six rats were included in the analyses shown throughout the article.

All animal procedures were conducted in accordance with the National Institutes of Health, International, and Institutional Standards for the Care and Use of Animals in Research and after consulting with a veterinarian.

### Experimental Rig

Each rat was trained in an operant box, equipped with: (1) a 21.5″ LCD monitor for presentation of the visual stimuli; (2) an array of three feeding needles, connected to three touch sensors for initiation of behavioral trials and collection of responses; and (3) two computer-controlled syringe pumps for automatic liquid reward delivery on the left-side and right-side feeding needles (see Alemi-Neissi et al., 2013 for further details). Rats learned to insert their head through a 4-cm diameter opening in the front wall of each box, so as to face the stimulus display and interact with the sensors' array. Constraining the head within such a viewing hole allowed its position to be largely reproducible across behavioral trials and very stable during stimulus presentation (see Alemi-Neissi et al., 2013 for a quantification), thus guaranteeing a tight control over the retinal size of the stimuli.

### Visual stimuli

The rats were trained to discriminate a pair of four-lobed visual objects that were transformed along a variety of dimensions (see below). Since the results of this study are compared with those of our previous study (Alemi-Neissi et al., 2013), where a different pair of objects was used, we have adopted the following naming convention to label individual objects, objects pairs, rats and groups of rats. We refer to the group of rats tested in our previous work as “group 1” (including rats numbered from 1 to 6), and to the pair of objects used in that study as “Stimulus Set 1,” containing Objects 1 and 2 (shown in Figure 1A, left panels). Conversely, we refer to the group of rats tested in the present study as “group 2” (including rats numbered from 7 to 9, given that only three animals succeeded in the discrimination task; see Section Subjects), and to the pair of objects used in this study as “Stimulus Set 2,” containing Objects 3 and 4 (shown in Figure 1B, left panels).

For both stimulus sets, the objects were renderings of three-dimensional models that were built using the ray tracer POV-Ray (http://www.povray.org/). Objects were rendered in a white, bright opaque hue against a black background. Each object's default size was 35° of visual angle (longest image dimension), and their default position was the center of the monitor.

Compared to Stimulus Set 1, the objects in Stimulus Set 2 were designed to be substantially more similar at the structural level. As such, the constituent parts of Objects 3 and 4 (i.e., three small ellipsoidal lobes attached to a large elliptical lobe; see Figure 1B, left panels) had a similar size, position, aspect ratio and overall layout. By contrast, the objects in Stimulus Set 1 were structurally quite dissimilar (see Figure 1A, left panels). Object 1 was made of a large, elliptical top lobe, attached to two smaller, overlapping bottom lobes, while Object 2 was composed of three elongated lobes that were approximately equally sized and equally spaced (radially). As a consequence, the overlap between Object 3 and 4 was larger than the overlap between Object 1 and 2 (see Figures 1A,B, rightmost panel), resulting in an overall larger pixel-wise similarity between the objects of Stimulus Set 2, as compared to Stimulus Set 1, across all tested views (see Results and Table 1 for details).

**Table 1:** **Normalized Euclidean distance between matching views of the objects within each Stimulus Set**.

	**Default**	**Size**	**Azimuth left**	**Azimuth right**	**Positions**	**In-plane rotations**
Object 1 and 2 (Stim. Set 1)	0.28	0.16	0.2	0.23	0.24	0.26
Object 2 and 3 (Stim. Set 2)	0.2	0.11	0.16	0.19	0.17	0.19

*The normalized, pixel-wise Euclidean distance between matching views the two objects in each stimulus set was computed for all the conditions tested with the bubbles masks*.

### Experimental design

#### Phase I: diagnostic features underlying recognition of the default object views

Rats were initially trained to discriminate the two default views of Objects 3 and 4 (Figure 1B, left panels). The animals learned: (1) to lick the central sensor, so as to trigger the presentation of one of the objects on the stimulus display; and (2) to lick either the right or left sensor, so as to report the identity of the currently presented object (see Figure 1C). Successful discrimination led to delivery of reward through the corresponding reward port/sensor, while failure to discriminate resulted in a time out period. The stimulus presentation time ranged between 2.5 and 4 s (see Alemi-Neissi et al., 2013 for further details).

Once a rat achieved ≥70% correct discrimination of the default object views (which typically required 3–12 weeks of training), a classification image method, known as the Bubbles (Gosselin and Schyns, 2001), was applied to identify what visual features were critical for the accomplishment of the task. This method consists in superimposing on a visual stimulus an opaque mask, containing a number of circular, semi-transparent openings, or *bubbles* (Figure 1E). An observer will be able to identify the stimulus only if the visual features that are diagnostic of its identity remain visible through the bubbles. This will allow inferring what image regions produced a positive (or, conversely, a negative) behavioral outcome.

**Figure 2:**
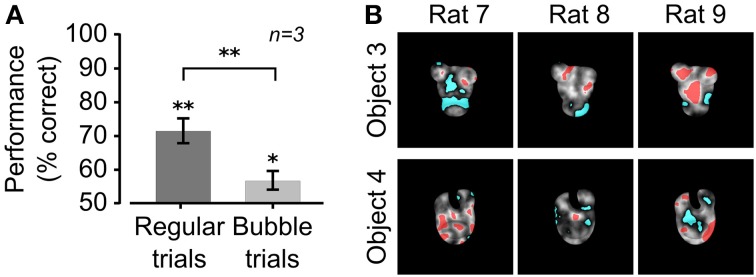
**Critical features underlying recognition of the default object views. (A)** Rat group average performance at discriminating the default object views was significantly lower in bubbles trials (light gray bar) than in regular trials (dark gray bar; *p* < 0.01; one-tailed, paired *t*-test), although both performances were significantly larger than expected by chance (^*^p < 0.05, ^**^p < 0.01; one-tailed, unpaired *t*-test). Error bars: SEM. **(B)** For each rat, the saliency maps resulting from processing the bubbles trials collected for the default object views are shown as grayscale masks superimposed on the images of the objects. The brightness of each pixel indicates how likely was, for an object view, to be correctly identified when that pixel was visible through the masks. Significantly salient and anti-salient object regions (i.e., regions that were, respectively, significantly positively or significantly negatively correlated with the correct identification of an object; *p* < 0.05; permutation test) are shown, respectively, in red and cyan.

In our implementation of the Bubbles method (see Alemi-Neissi et al., 2013, for details), the bubbles' size was fixed to 2° of visual angle, while their number was randomly chosen, in each trial, between 10 and 90, in steps of 20 (see examples in Figure 1F). This typically reduced the performance from ~65–75% correct obtained in unmasked trials to ~55–60% (see Figure 2A). Trials in which the default object views were shown unmasked (referred to as “regular trials”) were randomly interleaved with trials in which they were masked (referred to as “bubbles trials,” see Figure 1G). The fraction of bubbles trials in a daily session varied between 0.4 and 0.75. To obtain enough statistical power to extract the diagnostic features underlying rat recognition, at least 3000 bubbles trials per object were collected.

#### Phase II: diagnostic features underlying recognition of the transformed object views

**Figure 3:**
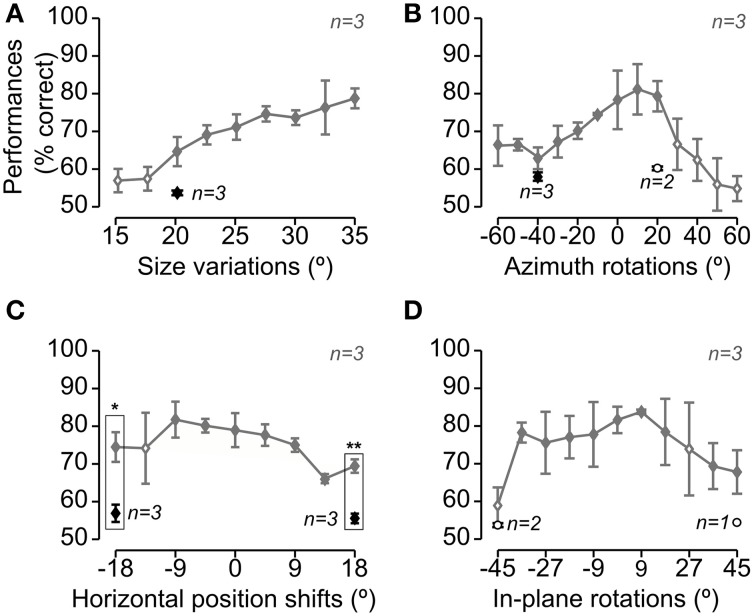
**Rat average recognition performance over the four dimensions along which the objects were transformed**. Gray and black symbols show performances in, respectively, regular and bubbles trials that were collected during the same sessions of Experimental Phase II (i.e., in interleaved regular and bubbles trials, as shown in Figure 1H). Panels **A–D** show the performances obtained, respectively, for size changes **(A)**, azimuth rotations **(B)**, translations **(C)** and in-plane rotations **(D)**. Solid and open diamonds indicate performances that were, respectively, significantly and non-significantly higher than chance (*p* < 0.05; one-tailed, unpaired *t*-test). Open circles refer to conditions (i.e., object views) for which less than 3 rats were tested with the Bubbles method (in this case, the significance of the performance was not tested). The rectangular frames refer to conditions in which the performance in regular trials was significantly larger than in bubbles trials (*p* < 0.05, one-tailed, paired *t*-test; again, only conditions for which all three rats were tested, in both regular and bubbles trials, were tested for significance). Error bars: SEM.

The animals were subsequently trained to tolerate variations in the appearance of the target objects along four different transformation axes (see Figure 1D), in the following order: (1) *size* variations, ranging from 35 to 15° visual angle; (2) *azimuth* rotations (i.e., in-depth rotations about the objects' vertical axis), ranging from −60 to 60°; (3) horizontal *position* changes, ranging from −18 to +18° visual angle; and (4) *in-plane* rotations, ranging from −45 to +45°. Each transformation was trained using an adaptive staircase procedure that is fully described in Alemi-Neissi et al. (2013) and Zoccolan et al. (2009). Once an animal had learnt to tolerate a wide range of variation along a given transformation axis (the extremes of each axis are shown in Figure 1D), one or more views along that axis were chosen, for each object, so that: (1) they were different enough from the default views of the two objects; and (2) most rats recognized them with a 60–70% correct performance (see Figure 3). These views (referred to as “bubbles views” in the following) were those selected for application of the Bubbles method and are highlighted by red frames in Figure 1D. Rats were then presented with randomly interleaved regular trials (in which unmasked objects could be shown across all the transformation axes trained up to that point) and bubbles trials (in which bubbles masks were superimposed to the bubbles views chosen from the most recently trained transformation; see an example of trial sequence in Figure 1H). As for the default object views, a minimum of 3000 bubbles trials was collected for each of the bubbles views. Note that, in general, for each rat, only some of the seven selected bubbles views could actually be tested, due to across-rat variation in life span and fluency in the invariant recognition task (see Alemi-Neissi et al., 2013, for details).

All experimental protocols were implemented using the freeware, open-source software package MWorks (http://mworks-project.org/). An *ad-hoc* plugin was developed in C++ to allow MWorks building bubbles masks and presenting them superimposed on the images of the visual objects.

### Data analysis

#### Computation of the saliency maps

A detailed description of the method for the extraction of the critical visual features underlying rat recognition of a given object view and the assessment of their statistical significance can be found in Alemi-Neissi et al. (2013). Briefly, this method consisted in two steps.

**Figure 4:**
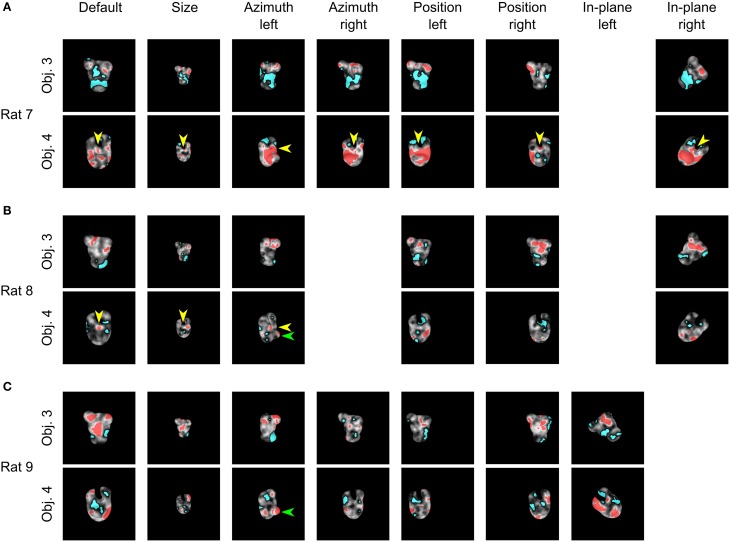
**Critical features underlying recognition of the transformed object views**. For each rat, the saliency maps that were obtained for each transformed view of Object 3 and 4 are shown. Red and cyan patches refer, respectively, to significantly salient and anti-salient regions (as in Figure 2B). The yellow arrows in **(A,B)** point to the salient feature located at the intersection between the two upper lobes of Object 4. This feature was repeatedly selected by both rats, either throughout all (rat 7) or the first three (rat 8) object views. The green arrows in **(B,C)** point at the salient feature located on the noise-like lobe of Object 4, which become fully visible only for the azimuth rotated view to the left, thereby affording the possibility to be used as a distinctive feature (as it happens for rat 8 and 9).

First, saliency maps were obtained that measured the correlation between the transparency values of each pixel in the bubbles masks and the behavioral responses. Throughout the article, these saliency maps are shown as grayscale masks superimposed to the images of the corresponding object views, with bright/dark pixels indicating regions that are salient/anti-salient, i.e., likely/unlikely to lead to correct identification of an object view, when visible through the bubbles masks (e.g., see Figures 2, 4). For a clearer visualization, the saliency values in each map were normalized by subtracting their minimum value, and then dividing by their maximum value.

As a second step, we computed which pixels, in a saliency map, had a statistically significant correlation with the behavior. To this aim, we performed a permutation test, in which the behavioral outcomes of bubbles trials were randomly shuffled (see Alemi-Neissi et al., 2013, for details). This yielded a null distribution of saliency values that was used to compute which values, in each saliency map, were significantly higher (or lower) than what obtained by chance (*p* < 0.05), and, therefore, which pixels, in the image, could be considered as significantly salient (or anti-salient). Throughout the article, significantly *salient regions* of an object view are shown in red, whereas *anti-salient regions* are shown in cyan (e.g., see Figures 2, 4).

Group average saliency maps and significant salient and anti-salient regions were obtained using the same approach, but after pooling the bubbles trials obtained for a given object view across all available rats (see **Figure 12**).

#### Ideal observer analysis

Rats' average saliency maps, as well as the maps obtained for individual rats, were compared to the saliency maps obtained by simulating a linear ideal observer (Gosselin and Schyns, 2001; Gibson et al., 2005; Vermaercke and Op de Beeck, 2012). Since this method is fully described in Alemi-Neissi et al. (2013), we provide here only a short, qualitative description.

Given a bubble-masked input image, the simulated observer classified it as being either Object 1 or 2, based on which of the eight views of each object (the templates), to which the mask could have been applied (shown by the red frames in Figure 1D), matched more closely the input image. The template matching was linear, since it consisted in computing a normalized dot product between each input images and each template. To better match rat retinal resolution, each input image was low pass-filtered, so that its spatial frequency content did not exceed 1 cycle per degree (i.e., the maximal resolving power of Long-Evans rats, Keller et al., 2000; Prusky et al., 2002). Finally, to lower the performance of the ideal observer and bring it close to rat performance, Gaussian noise (std = 0.5 of the image grayscale) was independently added to each pixel of the input images. Saliency maps and significant salient and anti-salient regions for the ideal observer were obtained as described above for the rats (see previous section).

Each rat saliency map (either individual or group averaged) was compared to the corresponding map obtained for the ideal observer by computing their Pearson correlation coefficient. The significance of the correlation was assessed by running a permutation test, in which the behavioral outcomes of the bubbles trials were randomly shuffled for both the rat and the ideal observer, so as to obtain a null distribution of correlation values, against which the statistical test was carried out at *p* < 0.05 (see Alemi-Neissi et al., 2013 for details).

#### Euclidean distance between matching views of the objects within each stimulus sets

To compare how similar were the objects belonging to a given stimulus set, we proceeded as follows. First, low pass-filtered versions of all the object views were produced, so that the spatial frequency content did not exceed the maximal retinal resolution of Long-Evans rats (i.e., 1 cycle per degree of visual angle). Then, we computed, within each stimulus set, the superposition of all the transformed views of both objects, and a crop rectangle was defined for each stimulus set as the minimal rectangle containing the resulting superposition. Next, a cropped version of each image (e.g., view) of the objects belonging to a given stimulus set was produced using the corresponding crop rectangle. The cropping was required to minimize the effect of uninformative black pixels surrounding the object views on the distance computations. Finally, the pixel-wise Euclidean distance between the cropped images of matching views of the two objects within a stimulus set was computed. This distance was then normalized to the maximal possible distance in the image space, which is the square root of the number of pixels (see Table 1). This allowed a fair comparison of object similarity between the two stimulus sets.

All data analyses were performed in Matlab (http://www.mathworks.com).

## Results

The goal of this study was to assess the influence of the structural similarity of the discriminanda on the adoption, by rats, of a view-based or a view-invariant recognition strategy. A group of rats (referred to as “group 2” throughout the article) was trained in an object recognition task that required the animals to discriminate two structurally (and visually) similar objects (i.e., Object 3 and 4, belonging to Stimulus Set 2, shown in Figure 1B, left panels). The results obtained from this group of rats were compared to those previously reported in a former study (Alemi-Neissi et al., 2013), where another group of rats (referred to as “group 1”) underwent the same training, but with objects that were more dissimilar at the structural level (i.e., Object 1 and 2, belonging to Stimulus Set 1, shown in Figure 1A, left panels). As in Alemi-Neissi et al. (2013), a classification image method, known as the Bubbles (Gosselin and Schyns, 2001), was applied to a subset of the trained object views to infer rat recognition strategy, and assess its reproducibility across views, as well as its consistency across subjects.

### Critical features underlying recognition of the default object views

During the initial experimental phase, the 6 rats of group 2 were trained to discriminate the default views of the objects belonging to Stimulus Set 2 (shown in Figure 1B, left panels). The training typically lasted 3–12 weeks before the animals achieved a criterion of ≥70% correct discrimination performance. Differently from the rats of group 1 (i.e., rats numbered from 1 to 6; see below and Alemi-Neissi et al., 2013 for details), only half of the animals (referred to as rat 7, 8, and 9 in the following) reached the criterion and were able to maintain it in the subsequent experimental phases. Once the criterion was reached, regular trials (i.e., trials in which the objects were shown unmasked) started to be randomly interleaved with bubbles trials (i.e., trials in which the objects were partially occluded by the bubbles masks; see Material and Methods for details and Figures 1E–H). By occluding parts of the visual objects, the bubbles masks made it harder for the rats to succeed in the discrimination task. In our experiments, we adjusted the number of the semi-transparent openings (the bubbles) in each mask, so as to bring each rat performance in bubbles trials to be ~10% lower than in regular trials. For the rats tested in this study (i.e., group 2, tested with Stimulus Set 2), the average recognition performance of the default views dropped from ~70% in regular trials to ~55% correct in bubbles trials (Figure 2A). The comparison with the rats tested in our previous study (i.e., group 1, tested with Stimulus Set 1), where the average recognition performance dropped from ~75% correct in regular trials to ~65% correct in bubbles trials (see Figure 3A in Alemi-Neissi et al., 2013), indicates that, as expected because of our stimulus design, objects in Stimulus Set 2 were harder to discriminate, especially when occluded by the bubbles masks.

The visual features underlying rat recognition strategy were extracted by measuring the correlation between bubbles masks' transparency values and rat behavioral responses (see Alemi-Neissi et al., 2013 for details). This yielded saliency maps, where the brightness of each pixel indicated the likelihood, for an object, to be correctly identified when that pixel was visible. Throughout the article, such saliency maps are displayed as grayscale masks superimposed on the images of the corresponding object views (see Figures 2B, 4, **12**). Saliency map values that were significantly higher or lower than expected by chance (*p* < 0.05, permutation test; see Materials and Methods) defined, respectively, significantly *salient* and *anti-salient regions* in the images of the object views (shown, respectively, as red and cyan patches in Figures 2B, 4, **12**). These regions are those objects' parts that, when visible through the masks, likely led, respectively, to correct identification and misidentification of the object views.

Contrarily to what found for Stimulus Set 1 (see Figure 3B in Alemi-Neissi et al., 2013), a larger inter-subject variability was observed in the saliency patterns obtained for the default views of the objects in Stimulus Set 2 (Figure 2B). In the case of Object 3, one or both the upper lobes were selected as salient features by all three rats of group 2. However, for rat 8, one of the features, the rightmost one, did not cover the upper right lobe. Rather, it was located slightly below it, at the margin of the central, largest lobe. This lobe, in turn, was mostly significantly salient for rat 9, but it was anti-salient for rat 7 (while, for the other two rats, anti-salient regions were located along the lower/right margin). In the case of Object 4, the top part of the central lobe was salient for two rats, in the guise of seven small, scattered patches for rat 7, and one single spot for rat 8. Interestingly, this spot, as well as one of the salient patches of rat 7, was located right at the curved-edge intersection between the central lobe and the top (smaller) lobes. On the other hand, for rat 9, this same part of the central lobe was anti-salient, along with the upper-right lobe. Similarly, the upper-right lobe was anti-salient for rat 7, while rat 8 showed spots of anti-saliency toward the right and the left margins of the object.

To summarize, although a few salient and anti-salient features were preserved across some of the rats (e.g., the top lobes of Object 3 and the small salient spot at the junction of Object 4's top and central lobes), a substantial inter-subject diversity was observed in terms of location, number, and size of the salient and anti-salient regions. This is indicative of the larger variety of perceptual strategies used by rats, when tested with structurally similar objects (such as the ones belonging to Stimulus Set 2), as compared to what we found using more dissimilar objects (such are those belonging to Stimulus Set 1, tested in Alemi-Neissi et al., 2013). These preliminary, qualitative observations will be quantified in the next sections.

### Critical features underlying recognition of the transformed object views

After being trained with the default views of Objects 3 and 4 and tested with bubble-masked versions of these views, the rats were further trained to recognize the objects in spite of transformations along four different variation axes: size, in-depth azimuth rotation, horizontal position and in-plane rotation. The tested ranges of variation are shown in Figure 1D, along with the views that, for each transformation axis, had been selected for application of the Bubbles method (referred to as “bubbles views” in the following; see red frames). The four transformation axes were trained sequentially, so that the amount of variation each rat had to tolerate increased gradually. In fact, the animals were confronted, at any given time during training/testing, with object views that were randomly sampled across all the variation axes tested up to that point (regular trials).

Similarly to what found for Stimulus Set 1 (see Figure 4 in Alemi-Neissi et al., 2013), also in the case of Stimulus Set 2, rat average recognition performance was significantly larger than chance for most of the tested object transformations, typically ranging from ~70 to ~80% correct and dropping below 70% correct only at the extremes of transformation axes, especially in the case of size changes and azimuth rotations (Figure 3, gray lines; see legend for details). Thus, in spite of their structural similarity, Objects 3 and 4 remained discriminable for the rats across a broad spectrum of image variation. On the other hand, the application of the bubbles masks resulted in a decrement of the recognition performance (see black diamonds) that was larger than the one observed in the case of Stimulus Set 1 (compare to Figure 4 in Alemi-Neissi et al., 2013). The average performance on bubbles trials ranged between 55 and 60% correct and was significantly below the performance observed in regular trials in the case of the translated object views (*p* < 0.05, one-tailed, paired *t*-test; see rectangular frames in Figure 3C), although it was still significantly above chance for all those transformations in which all three rats were tested (*p* < 0.05, one-tailed, unpaired *t*-test, see filled black diamonds; for the conditions tested with only 2 rats, significance was not assessed, see open circles). This suggests that, for rats, it was challenging to discriminate structurally similar objects, especially when shape information was degraded by reducing the size of the objects (see Figure 3A) or rotating/shifting them of large amounts (see Figures 3C,D), and simultaneously adding the semi-transparent bubbles masks.

Bubbles trials were analyzed as described in the previous section (see also Materials and Methods) to obtain saliency maps with highlighted significantly salient and anti-salient regions for each of the selected bubbles views (see Figure 4). A qualitative comparison between these saliency patterns and those previously obtained for the objects of Stimulus Set 1 (see Figure 6 in Alemi-Neissi et al., 2013) allows appreciating how rat recognition strategy depends on the structural complexity and visual similarity of the discriminanda.

Both Object 3 and 4 in Stimulus Set 2, just like Object 1 and 2 in Stimulus Set 1, were made of ellipsoidal structural parts (or lobes; see Figures 1A,B, left panels). However, in the case of Stimulus Set 2, such parts were less protruded and, more importantly, matching lobes in the two objects had a similar size, position and aspect ratio. Hence, they were less diagnostic of object identity, compared to the lobes of Objects 1 and 2, resulting in a larger similarity between the objects of Stimulus Set 2, as compared to Stimulus Set 1, across all tested views (see Table 1 for details). Consistent with this observation, we found a general tendency, for the diagnostic features of Object 3 and 4, to be distributed (often in a quite scattered way) over a region of the objects (i.e., top or bottom half) encompassing multiple lobes, rather than being precisely (and reproducibly) located in specific lobes (or lobes' sub-regions), as previously found for Objects 1 and 2 (see Alemi-Neissi et al., 2013). Nonetheless, we could still find, albeit less systematically as compared to Stimulus Set 1, a tendency to select (and, to some extent, “track” throughout different transformations) discrete object features (see yellow arrows in Figure 4 and the description below).

For rat 7, the salient features were located in the upper region of Object 3 for all tested conditions (Figure 4A, upper row), although, in the case of the default view, they were smaller, more scattered and mixed with anti-salient patches, which only remained as smaller spots in the azimuth-rotated views. The anti-salient regions covered preferentially the central and lower parts. A somewhat reversed pattern was observed for Object 4 (Figure 4A, lower row): the central/bottom region was largely salient across all tested views, starting with a combination of small patches in the default view, which reduced to a few small spots in the size-transformed condition, and finally merged into a big salient region for most of the remaining transformations. Interestingly, the salient spot located right at the intersection between the central lobe and the top lobes (see yellow arrows) was observed not only in the case of the default view (see previous section), but, systematically, across all tested conditions, either as a discrete feature or merging with the bigger salient patch.

Similarly to rat 7, rat 8 displayed a preference for the upper region of Object 3 in all tested conditions (Figure 4B, upper row). The anti-salient features generally covered the lower lobe, but extended to the central part of the object in three conditions (size transformed and horizontally shifted views) and to the upper-right lobe in one condition (horizontally shifted to the left). It was again the central part of Object 4 its most salient region (Figure 4B, lower row), but the salient patches remained small, few and scattered, and always mixed with anti-salient spots. Noticeably, also for rat 8, the intersection between the central lobe and the top lobes contained a small, significantly salient spot in the case of the default, azimuth-rotated and size-transformed views (see yellow arrows). This spot was also salient for the horizontally shifted views, although it did not cross the threshold for significance.

Compared to the previous two rats, rat 9 displayed, at the beginning (i.e., for the default views), a strategy that was more consistent with the selection of the discrete, constituent elements of the objects, rather than wide regions encompassing multiple lobes. For instance, the salient patches obtained for the default view of Object 3 (Figure 4C, upper row) matched closely the central lobe and the two upper lobes of the object. Although these discrete features did not remain salient for all the tested transformations, they were preserved in several of the subsequently tested views. In the case of Object 4 (Figure 4C, lower row), a more variegate combination of salient features (often mixed with anti-salient spots) was found across the tested views, covering both upper and lower regions of the object, although discrete lobes were still occasionally selected as salient features. One of these lobes was the bottom one (with a nose-like shape), which emerged as a salient feature in one condition (the azimuth rotated view to the left; see green arrow), i.e., when it became more protruded, as compared to all other views, and, therefore, more likely to be parsed by the rat visual system. This was observed also for rat 8 (Figure 4B, lower row, green arrow), although the salient spot was smaller.

To summarize, when facing objects that were hard to discriminate (as in the case of Stimulus Set 2), rats appeared to rely on a set of object features that was only partially preserved across transformations. While the overall object regions (i.e., either top or bottom half) containing either the salient or anti-salient patches tended to be preserved across different views, the size, number, and location of these patches varied substantially across conditions and rats. This result is in contrast with what found in our previous study for Object 1 and 2, where the salient features tended to be reproducibly located in specific positions of the objects' structural parts (e.g., the tips of the elongated lobes defining Object 2). In other words, rats tested with Stimulus Set 2, differently from those tested with Stimulus Set 1, did not show a strong, view-invariant preference for well-defined structural elements of the objects. These qualitative observations are quantified in the next sections, starting with the reproducibility of the patterns of salient features across object views.

### Is rat invariant recognition more consistent with a view-invariant or a view-based processing strategy?

**Figure 5:**
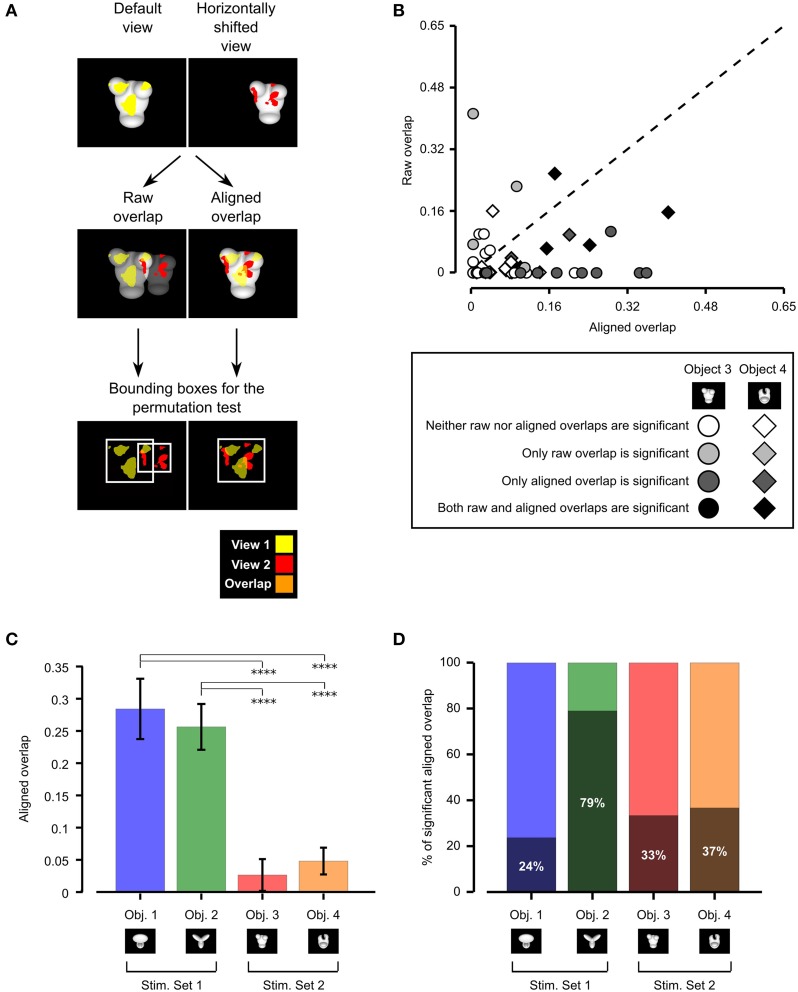
**Consistency of rat recognition strategy across object views. (A)** Illustration of the procedure to compute the *raw* and *aligned* overlap between the salient features' patterns obtained for two different views of an object. The default and the leftward horizontally shifted views of Object 3 are used as examples (first row). The raw features' overlap was computed by superimposing the images of the two object views (and the corresponding features' patterns) within the stimulus display (second row, left plot). The aligned features' overlap was computed by reversing the transformation that produced the leftward horizontally shifted view. That is, the object was shifted to the right of 18° and scaled back to 35°, so as to perfectly overlap with its default view (second row, right plot). In both cases, the overlap was computed as the ratio between the orange area and the sum of the red, yellow and orange areas. The significance of the overlap was assessed by randomly shifting the salient regions of each object view within the minimum bounding box (see white frames in the third row of the figure) enclosing each view. **(B)** For each pair of views of Object 3 (circles) and Object 4 (diamonds) resulting from affine transformations (i.e., position/size changes and in-plane rotations), the raw features' overlap is plotted against the aligned features' overlap. The shade of gray indicates whether the raw and/or the aligned overlap for a given view was significantly larger than expected by chance (*p* < 0.05; see caption). **(C)** Median aligned overlaps for the objects belonging to Stimulus Set 1 and 2. The error bars are standard errors of the medians (obtained by bootstrapping). The statistical significance of the difference between a given pair of medians was assessed by a Mann–Whitney *U*-test (^****^p < 0.0001). **(D)** Percentage of significant aligned overlap values for the objects belonging to two stimulus sets.

To quantify to what extent rat recognition of Objects 3 and 4 was consistent with a view-invariant visual processing strategy, we measured the overlap between the patterns of salient features obtained for all possible pairs of object views produced by affine transformations (i.e., all tested object views with the exclusion of in-depth azimuth rotations). This overlap was computed after reversing (i.e., “undoing”) the transformations that originated a pair of object views, so as to perfectly align one view on top of the other (e.g., in the case of the comparison between the default and the horizontally translated views shown in Figure 5A, the latter was shifted back to the center of the screen and scaled back to 35°, so as to perfectly overlap with the default view; see second row of Figure 5A, right panel). This procedure yielded *aligned* overlap values between pairs of salient features' patterns, which could be compared to those obtained for Objects 1 and 2 in Alemi-Neissi et al. (2013). Consistently with our previous study, we also computed, for each pair of views, *raw* overlap values, which quantified the amount of overlap between the salient features' patterns of two object views within the stimulus display (i.e., in absolute screen coordinates; see second row of Figure 5A, left panel). When plotted one against the other (Figure 5B), the aligned and the raw overlaps measured whether rat recognition was more consistent with a view-invariant strategy (in which the same set of object-centered features is relied upon and “tracked” across different views) or a screen-centered strategy (i.e., a low-level strategy, where one or more image patches exist, at specific locations within the stimulus display, that remain diagnostic of object identity in spite of view changes, thus affording a trivial solution to the invariant recognition task).

Following Nielsen et al. (2006), both the aligned and raw overlaps were computed as the ratio between overlapping area and overall area of the significantly salient regions of the two object views under comparison (e.g., as the ratio between the orange area and the sum of the red, yellow, and orange areas in Figure 5A, second row). As done in our previous study (Alemi-Neissi et al., 2013) and in Nielsen et al. (2006), the significance of each individual raw and aligned overlap was assessed at *p* = 0.05 through a permutation test (1000 permutation loops), in which the salient regions of each object view in a pair were randomly shifted within the minimum bounding box enclosing each view (see Figure 5A, bottom row and Alemi-Neissi et al., 2013, for details).

As shown by the scatter plot in Figure 5B, for about 62% of the tested view pairs (i.e., in 37 out of 60 cases), the aligned overlap was larger than the raw overlap. Although this proportion was much higher for the objects belonging to Stimulus Set 1, as assessed in our previous study (i.e., about 92% of view pairs had a larger aligned overlap; see Figure 8B in Alemi-Neissi et al., 2013), Figure 5B shows that, also for Stimulus Set 2, a trivial, screen-centered strategy could not explain rat recognition behavior. This conclusion was confirmed by the fact that, for both objects belonging to Stimulus Set 2, the average aligned overlap values were significantly higher than the raw values (Object 3: aligned 0.09 ± 0.02 vs. raw 0.04 ± 0.02, *p* < 0.05; Object 4: aligned 0.07 ± 0.02 vs. raw 0.03 ± 0.01, *p* < 0.01; significance was assessed through a paired permutation test, in which the sign of the difference between aligned and raw overlap for each pair of views was randomly assigned in 10,000 permutation loops). In addition, for both objects, the number of cases in which the aligned overlaps were larger than expected by chance was approximately twice as large as the number of significant raw overlaps—for Object 3, 10/30 aligned vs. 5/30 raw overlaps were significant, while, for Object 4, 11/30 aligned vs. 7/30 raw overlaps were significant (see Figure 5B, where significance is coded by the shade of gray filling the symbols).

To better understand the influence of object structure on the adoption of a view-invariant strategy, we reported side by side in Figure 5C the median aligned overlaps obtained for the objects tested in our previous study (i.e., Objects 1 and 2, Stimulus Set 1) and in the current one (i.e., Objects 3 and 4, Stimulus Set 2). The resulting bar chart shows that the aligned overlap was much larger for the objects belonging to Stimulus Set 1, as compared to the objects of Stimulus Set 2 (and this difference was significant at *p* < 0.0001, Mann–Whitney *U*-test). In addition, for Object 2, the large majority of aligned overlap values was significantly higher (79%) than expected by chance, while, for the other objects, the percentage of significant overlaps ranged from 24 to 37% only (see Figure 5D). This implies that the pattern of salient features was much more reproducible for the objects belonging to Stimulus Set 1, as compared to Stimulus Set 2, and, in particular, for Object 2, which was the object made of the more distinctive structural parts (as discussed at length in Alemi-Neissi et al., 2013). To summarize, Figure 5 quantifies the qualitative observations of the previous section—the larger was the discriminability of the visual objects (as in the case of Stimulus Set 1) and the more distinctive were their structural elements (as in the case of Object 2), the more view-invariant was rat recognition strategy (i.e., the animals consistently used the same structural parts of the objects, across different views, as diagnostic features of object identity). For the less discriminable objects (i.e., Stimulus Set 2), rat recognition strategy was still more consistent with an object-based tracking of broadly defined saliency regions (e.g., the top or bottom parts of the stimuli) than with a low-level, screen-centered detection of transformation-preserved diagnostic image spots. However, the specific patterns of salient features were much more view-dependent than in the case of Stimulus Set 1 (and of Object 2 in particular).

To quantitatively assess whether the difference between the strategies used by the two groups of rats could be attributed to objects' similarity, we computed the normalized, pixel-wise Euclidean distance between matching views of the objects within each stimulus set (see Materials and Methods). Only the views on which the bubbles masks were applied (i.e., the bubbles views) were considered in this analysis. The result of this comparison is reported in Table 1. As expected, the distance between the views of the objects belonging to Stimulus Set 1 was systematically larger than the distance between the views of the objects belonging to Stimulus Set 2. This resulted in an average pixel-level discriminability that was significantly higher for Stimulus Set 1, as compared to Stimulus Set 2 (0.23 ± 0.02 vs. 0.17 ± 0.01, respectively; one-tailed, paired *t*-test, *p* < 0.001).

### Comparing the compactness of the salient features' patterns among stimulus sets and individual objects

**Figure 6:**
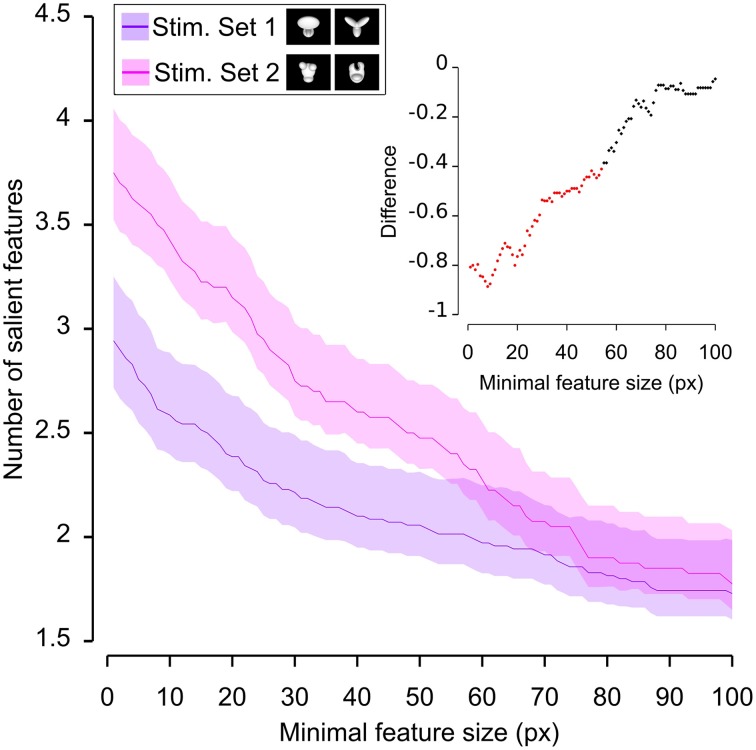
**Average number of salient features obtained for the two stimulus sets**. The average number of salient features obtained for the view of an object belonging to either Stimulus Set 1 or 2 (see the caption for the color code) is plotted as a function of the minimal size of the features that were taken into account for this analysis (the average was computed by pooling across all views of both objects within a stimulus set and all rats). The difference between the values obtained for two stimulus sets is plotted in the inset as a dotted line, where the color codes its significance—black, no significant difference; red, significant difference at *p* < 0.05 (two-tailed, unpaired *t*-test).

**Figure 7:**
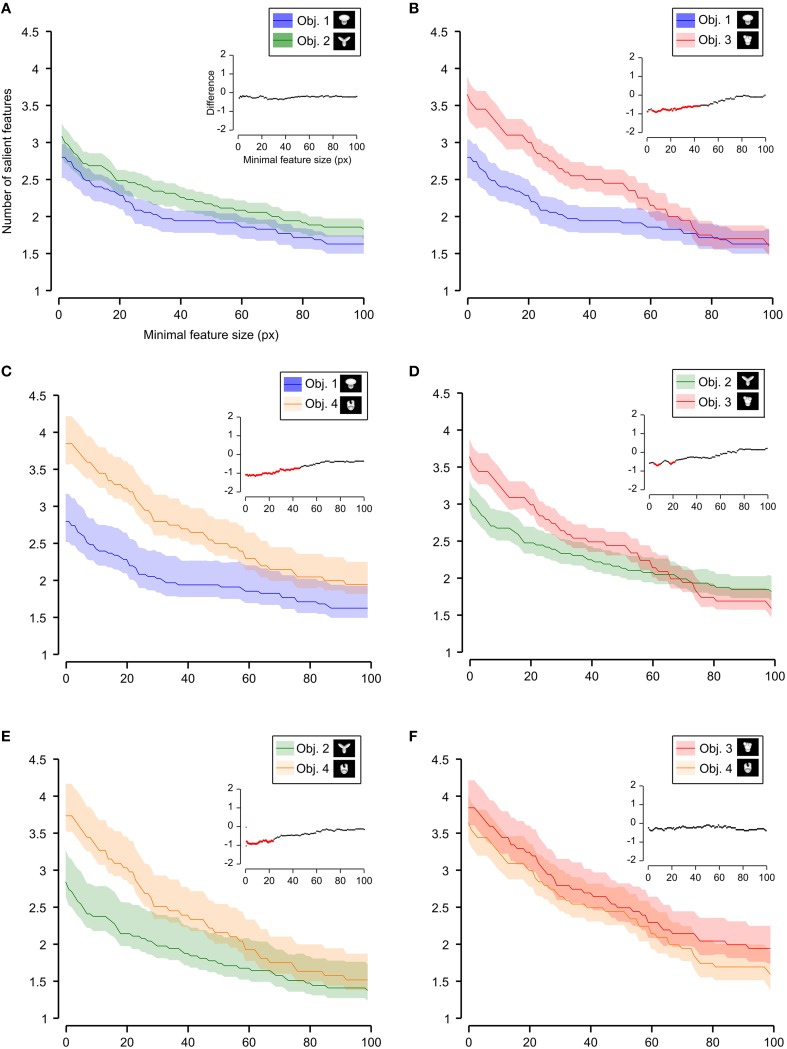
**Average number of salient features obtained for individual objects**. The average number of salient features obtained for the view of an object is compared for each possible pair of objects (object identity is color coded in **(A–F)**; see caption on the top of each panel). The shaded regions are SEM. The average was computed by pooling across all views of an object and all rats, and was plotted against the minimal size of the features that were taken into account for this analysis. The insets show the difference between the values obtained for each objet pair (same color code as in Figure 6—black, no significant difference; red, significant difference at *p* < 0.05; two-tailed, unpaired *t*-test).

**Figure 8:**
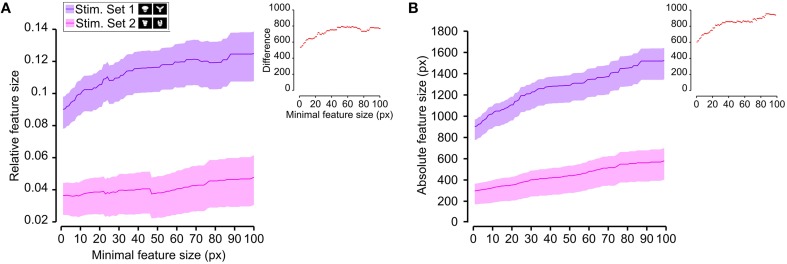
**Average relative and absolute size of the salient features obtained for the two stimulus sets**. The average size of the salient features obtained for the views of the objects belonging to either Stimulus Set 1 or 2 (see the caption for the color code) is plotted as a function of the minimal size of the features that were taken into account for this analysis (the average was computed by pooling across all features, all views of both objects within a stimulus set and all rats). The shaded regions are SEM. **(A,B)** show, respectively, the relative and absolute feature size (with the former computed by dividing the size of each feature by the overall area of the corresponding object view). The insets show the difference between the values obtained for the two stimulus sets (same color code as in Figures 6, 7—black, no significant difference; red, significant difference at *p* < 0.05; two-tailed, unpaired *t*-test).

**Figure 9:**
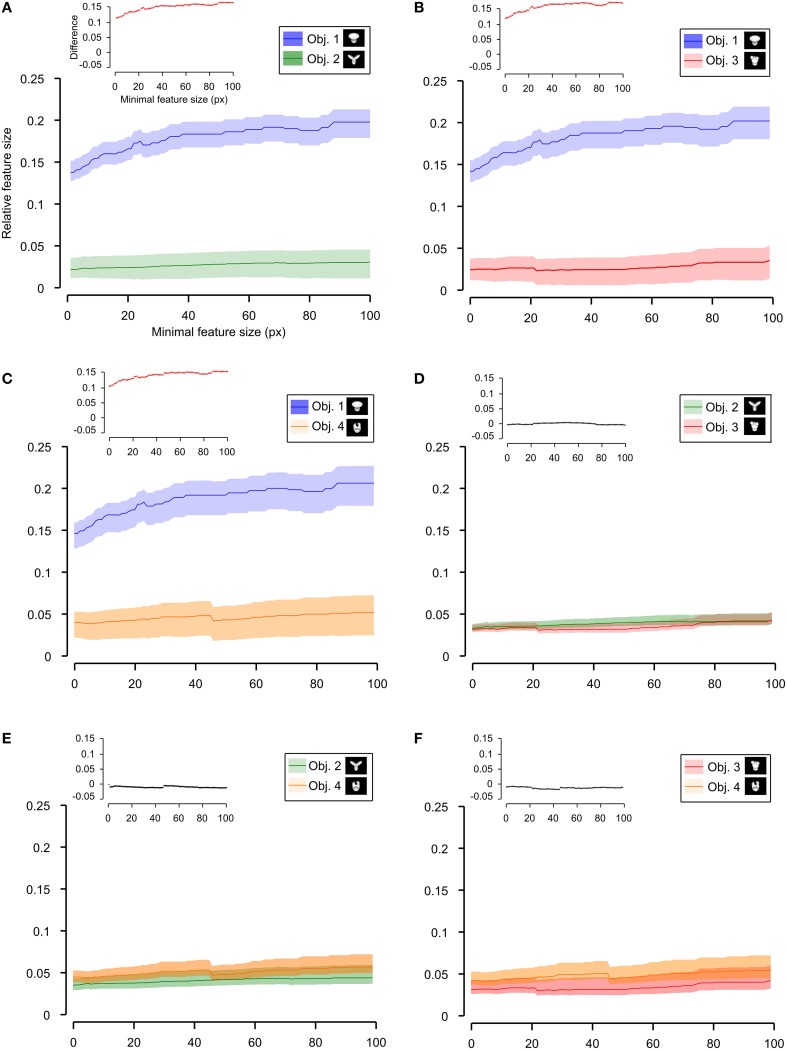
**Average relative size of the salient features obtained for individual objects**. The average relative size of the salient features obtained for the views of an object is compared for each possible pair of objects [object identity is color coded in **(A–F)**; see caption on the top of each panel]. The shaded regions are SEM. The average was computed by pooling across all features, all views of an object and all rats, and was plotted against the minimal size of the features that were taken into account for this analysis. The relative size was computed as described in Figure 8. The insets show the difference between the values obtained for each objet pair (same color code as in Figures 6–8—black, no significant difference; red, significant difference at *p* < 0.05; two-tailed, unpaired *t*-test).

**Figure 10:**
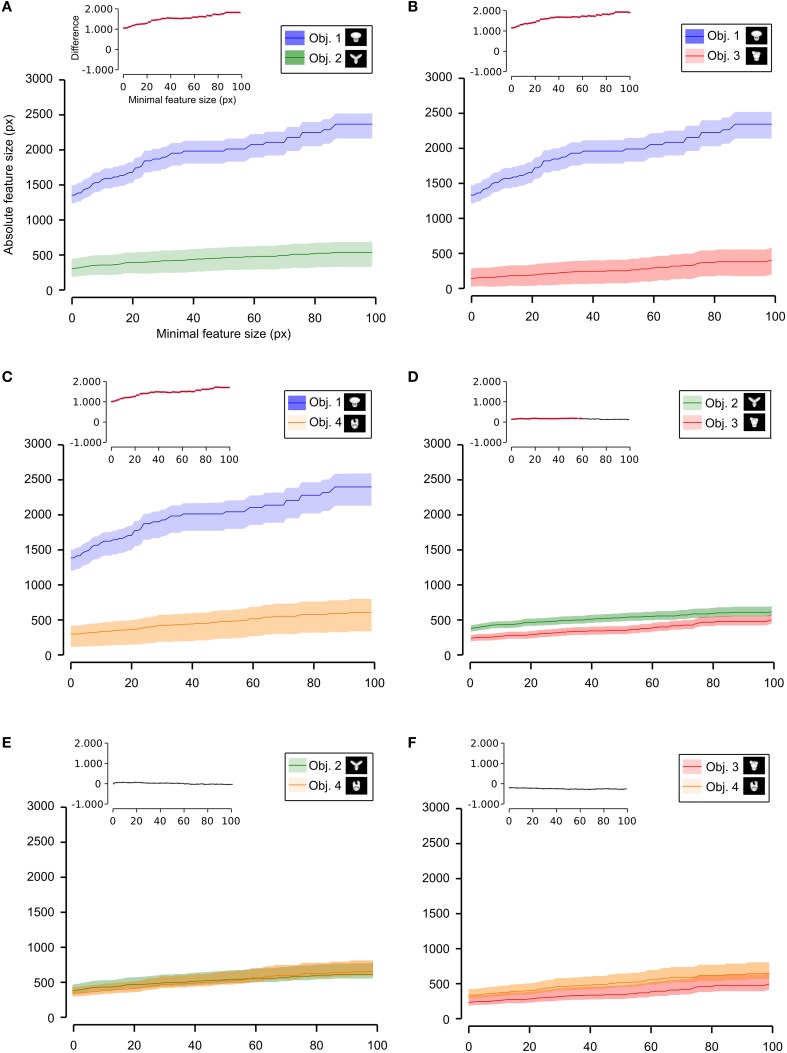
**Average absolute size of the salient features obtained for individual objects**. The average absolute size of the salient features obtained for the views of an object is compared for each possible pair of objects [object identity is color coded in **(A–F)**; see caption on the top of each panel]. The shaded regions are SEM. The average was computed by pooling across all features, all views of an object and all rats, and was plotted against the minimal size of the features that were taken into account for this analysis. The insets show the difference between the values obtained for each objet pair (same color code as in Figures 6–9—black, no significant difference; red, significant difference at *p* < 0.05; two-tailed, unpaired *t*-test).

Having quantified the different discriminability of the two object pairs, we further assessed how such a difference affected the recognition strategy of the two groups of rats by comparing the average number (Figures 6, 7) and the average absolute and relative size (Figures 8–**10**) of the salient features found for each object (with the average taken across all tested bubbles views). Since the absolute size of the salient features ranged from a few pixels (in the case of spot-like features) to hundreds of pixels (in the case of features spanning over large fractions of the objects; see Figure 4 and also Figure 6 in Alemi-Neissi et al., 2013), we measured how these quantities (e.g., the number of salient features) varied when only features having a size larger than a minimal threshold value (ranging from 1 to 100 pixels) were taken into account. We then assessed, at each threshold value, the statistical significance of the difference between (e.g.) the average number of features obtained, across all tested views, for the two stimulus sets (two-tailed, unpaired *t*-test at *p* < 0.05; see Figure 6, where the red traces in the inset show the comparisons yielding a significant difference). The same analysis was carried out for each of the six possible pairs of objects belonging to the two object sets (e.g., see Figure 7 for the comparison regarding the number of salient features).

We found that the average number of salient features was larger for Stimulus Set 2 (Figure 6, pink line) than for Stimulus Set 1 (Figure 6, purple line) and this difference was significant over a large range of minimal feature sizes (from 1 to about 55 pixels; see red dots in the inset of Figure 6). Only asymptotically (for very large feature sizes), the difference between the numbers of features found for the two stimulus sets became not significant (see black dots in the inset of Figure 6). This is expected, given that, by construction, only a few large features covering big portions of the objects are left, regardless of the stimulus set, when the minimal feature size is very large. Focusing on individual objects (Figure 7), i.e., considering all possible pairs of the four objects (regardless whether an object belonged to Stimulus Set 1 or 2), we found that the average number of salient features for Object 1 was significantly smaller than for Object 3 and 4 (Figures 7B,C), as long as the minimal feature size did not cross the 45–50 pixel value (see insets), while it was never significantly different from the number of salient features of Object 2 (Figure 7A). Object 2 displayed a smaller difference, in terms of number of features, when compared to object 4 (significant up to a minimal feature size of ~20 pixels; see Figure 7E), and even smaller when compared to Object 3 (significant in the ranges of minimal feature size between 5–10 and 18–22 pixels; see Figure 7D). No significant difference was found between Object 3 and 4 (Figure 7F).

Next, we computed the size of the salient features obtained for the four objects across all the views that were tested with the bubbles masks. For each object view, we measured the absolute size (in pixels) of all the salient features obtained for that view. Then, the features' sizes obtained for all the views were pooled to obtain the average absolute feature sizes shown in Figures 8B, **10**. Using the same approach, we also computed the average relative feature sizes shown in Figures 8A, 9. The only difference was that, in this case, the size in pixels of each salient feature was divided by the overall area (in pixels) of the corresponding object view, thus yielding the portion of the view that was covered by that feature.

As shown in Figure 8, a comparison between the two stimulus sets revealed that the rats tested with the objects belonging to Stimulus Set 1 selected, on average, larger features, compared to the rats tested with Stimulus Set 2, in terms of both absolute and relative size. This difference was significant for every minimal feature size under consideration (two-tailed, unpaired *t*-test at *p* < 0.05; see red dots in the insets of Figure 8). However, when we considered the differences between individual object pairs, in terms of their features' relative size (Figure 9), we found that the only significant difference was between Object 1 and all the other objects (see Figures 9A–C). When the absolute size values were compared (Figure 10), a significant difference was also observed between Object 2 and Object 3 (Figure 10D).

Taken together, the analyses shown in Figures 6–10 revealed a tendency for the salient features' patterns obtained for Objects 1–4 to closely match the distinctiveness and prominence of the objects' structural parts. For objects with large, clearly discriminable lobes (such as the top lobe of Object 1 and the three elongated lobes of Object 2), the diagnostic salient features were more compact (i.e., larger and less numerous). Objects with smaller and less distinctive lobes (such as Objects 3 and 4) displayed a more scattered pattern of salient features (i.e., smaller and more numerous salient patches). Not surprisingly, this difference in the compactness of the salient features was more prominent when Object 1 (the object with the largest and most distinctive lobe) was compared to the objects of Stimulus Set 2. Once again, this finding suggests that rat recognition strategy is strongly dependent on the structural properties of the target objects.

### Between-subject reproducibility of rat recognition strategy

**Figure 11:**
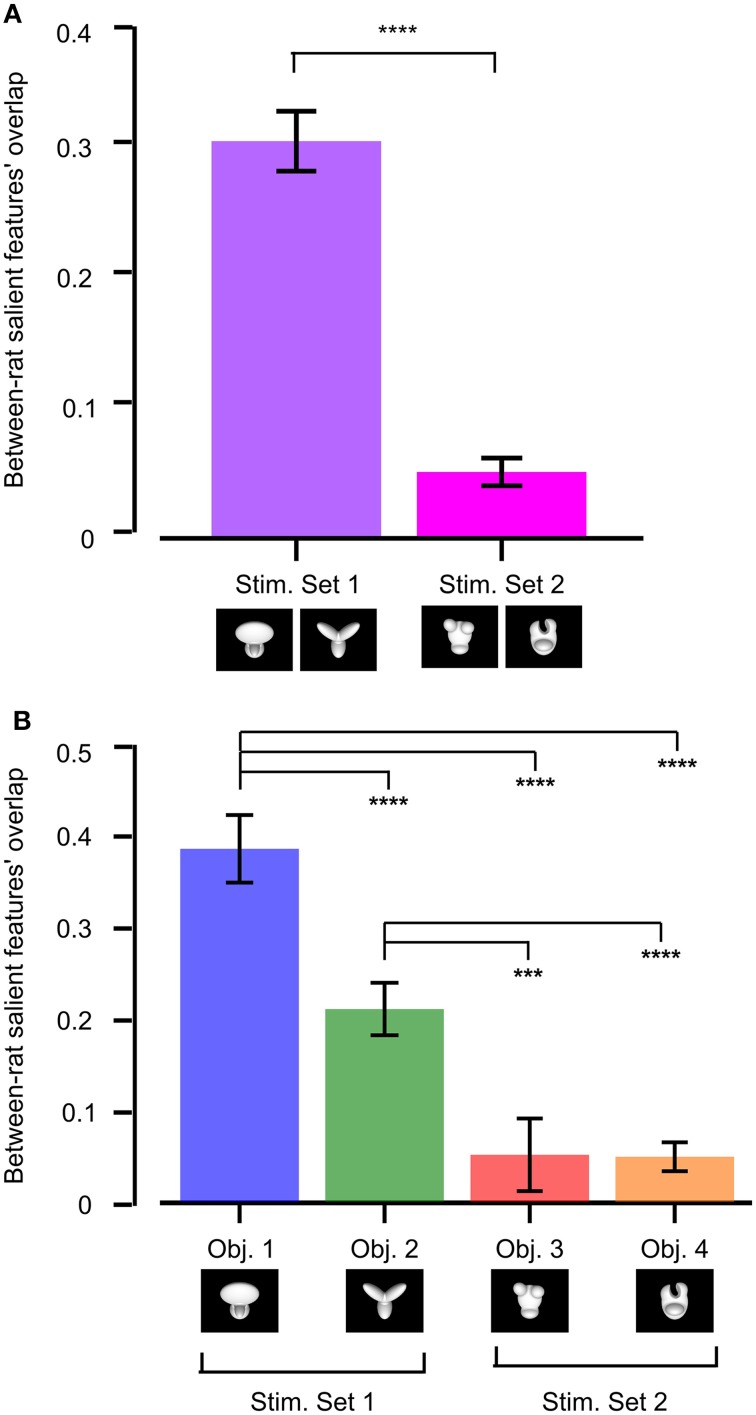
**Between-rat consistency of the recognition strategy. (A)** Between-rat consistency of the salient features' patterns obtained for the objects belonging to Stimulus Set 1 and 2. For any given object view, the overlap between the pattern of salient features obtained for two different rats was computed. Overlap values obtained for all the views of the objects within a stimulus set and all possible pairs of rats were polled, yielding the median overlaps per stimulus set shown by the colored bars. **(B)** Same analysis as in **(A)**, but with the overlap values of individual objects considered independently. In both **(A,B)**, a Mann–Whitney *U*-test was applied to check whether the resulting medians were significantly different from each other (^***^p < 0.001, ^****^p < 0.0001).

To quantify whether stimulus discriminability also affected the reproducibility of the object features that were preferentially chosen by one group of rats (tested with the same object conditions), we measured the across-rat consistency of the salient features' patterns obtained for our two stimulus sets. This was achieved by computing the overlap of the pattern of salient features obtained for one rat at a given object view (e.g., the default view) with the pattern of salient features obtained for another rat at the same object view (the overlap was computed in the same way as described in Figure 5). All possible views and all possible rat pairs were considered to obtain the resulting median overlap values shown in Figure 11.

The median overlap was much larger for Stimulus Set 1 than for Stimulus Set 2, and such a difference was highly significant (*p* < 0.0001, Mann–Whitney *U*-test test; see Figure 11A). When the results of individual objects were compared, Object 1 displayed the largest between-rat consistency of the salient features selected to solve the task, followed by Object 2 and then by the objects belonging to Stimulus Set 2, with all the pairwise comparisons, except the one between Object 3 and 4, yielding differences that were significantly larger than expected by chance (*p* < 0.001; Mann–Whitney *U*-test test; see Figure 11B). This confirms the observation that the rats tested with Stimulus Set 2 used a recognition strategy that was much more consistent with a view-dependent selection of object features, with respect to the rats tested with Stimulus Set 1, as noticeable by comparing Figure 4 to Figure 6 in Alemi-Neissi et al. (2013). It also confirms that, within Stimulus Set 1, the object leading to the most consistent selection of same diagnostic features was the one that, having the simplest structure (i.e., Object 1), afforded one single feature (the top, large lobe) for its identification. Object 2, with its equally sized and equally distinctive lobes, allowed a larger number of perceptual alternatives (i.e., possible feature combinations) for its recognition. Hence, the slightly (but significantly) lower between-rat consistency observed for Object 2, as compared to Object 1. However, since each individual feature was reproducibly confined to the tip of one of the lobes, and, in most cases, at least two lobes were used by rats as diagnostic features, Object 2 still displayed a pattern of features that was much more consistent, across rats, than what we found for the objects of Stimulus Set 2.

### Comparison between the saliency maps obtained for the rats and a simulated ideal observer

**Figure 12:**
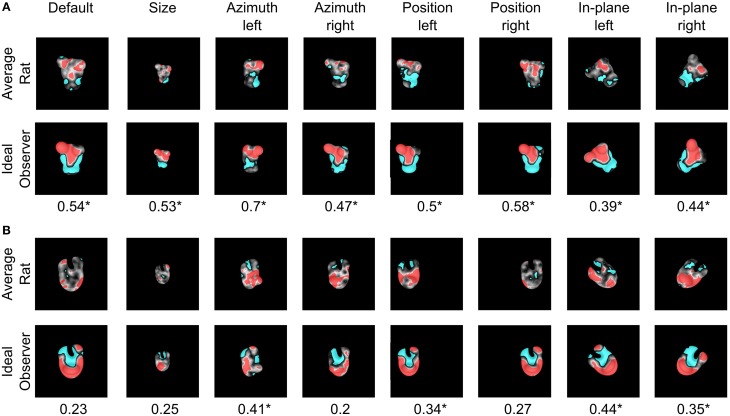
**Critical features' patterns obtained for the average rat and a simulated ideal observer**. Rat group average saliency maps obtained for Objects 3 **(A)** and 4 **(B)**, with highlighted significantly salient (red) and anti-salient (cyan) features (top rows), are compared to the saliency maps obtained for a simulated ideal observer (bottom rows). Below each object view, the Pearson correlation coefficient between the saliency maps obtained for the average rat and the ideal observer is reported. The significance of the correlation was assessed by a permutation test (^*^p < 0.05).

The finding that rat recognition strategy is more or less view-invariant, depending on the level of stimulus discriminability, raises the question of how optimal such a strategy was, given the discriminatory information that each pair of visual objects afforded. To address this question, we compared it to the strategy of a simulated ideal observer that was tested using the same bubble-masked images that had been presented to the rats of both experimental groups. Given a stimulus set (i.e., either Stimulus Set 1 or 2), the simulated observer performed a template-matching operation between incoming bubble-masked input images and each of the possible bubbles views of the objects within the set (e.g., those marked by red frames in Figure 1D), to find out to what object each input image corresponded to. The simulated observer was *ideal*, since it had stored in memory, as templates, all the views that each object within the stimulus set could take, and was *linear*, because the template-matching operation consisted in computing the dot product between each input image and each template view (see Materials and Methods and Alemi-Neissi et al., 2013, for details). The simulated observer could be incorrect or correct in identifying the object in a given bubble-masked input image, depending on whether the mask occluded parts of the object that were more or less diagnostic of its identity. Analyzing the responses of the ideal observer to the different bubble-masked images yielded saliency maps that were analog (and, therefore, directly comparable) to the ones previously obtained for the rats. Specifically, the saliency maps obtained for the ideal observer were compared both with the maps obtained for the individual rats (see Table 2) and with the group average maps that were obtained by pooling the bubbles trials collected for a given object view across all available rats that had been tested with that view (see Figure 12, and, by comparison, Figure 10 in Alemi-Neissi et al., 2013).

**Table 2:** **Comparison between the saliency maps obtained for the rats and a simulated ideal observer**.

	**Default**	**Size**	**Azimuth left**	**Azimuth right**	**Position left**	**Position right**	**In-plane left**	**In-plane right**
**Obj.1**								
Rat 1	0.08	0.28^*^	−0.20	0.06	0.22	0.27	/	/
Rat 2	0.25^*^	0.36^*^	−0.17	0.06	0.3^*^	−0.09	0.22^*^	/
Rat 3	0.18	0.57^*^	−0.22	0.22	0.3^*^	0.19	0.24	0.51^*^
Rat 4	0.08	0.52^*^	−0.13	/	0.33^*^	/	/	/
Rat 5	0.25^*^	0.43^*^	−0.13	0.03	/	/	/	/
Rat 6	−0.1	0.35^*^	−0.04	0.09	0.19	/	/	/
**Obj.2**								
Rat 1	0.48^*^	0.34^*^	0.46^*^	0.4^*^	0.26	0.54	/	/
Rat 2	0.51^*^	0.32^*^	0.5^*^	0.57^*^	0.45^*^	0.39^*^	0.55^*^	/
Rat 3	0.55^*^	0.37^*^	0.43^*^	0.44^*^	0.36^*^	0.55^*^	0.62^*^	0.59^*^
Rat 4	0.33^*^	0.15	0.35^*^	/	0.4^*^	/	/	/
Rat 5	0.54^*^	0.26	0.43^*^	0.46^*^	/	/	/	/
Rat 6	0.41^*^	0.45^*^	0.12	0.36^*^	0.47^*^	/	/	/
**Obj.3**								
Rat 7	0.25	0.34^*^	0.54^*^	0.5^*^	0.33^*^	0.3	/	0.39^*^
Rat 8	0.33^*^	0.46^*^	0.48^*^	/	0.44^*^	0.55^*^	/	0.42^*^
Rat 9	0.49^*^	0.37^*^	0.57^*^	0.1	0.48^*^	0.37^*^	0.39^*^	/
**Obj.4**								
Rat 7	0.07	0.25	0.27	0.17	0.23	0.06	/	0.3^*^
Rat 8	−0.33^*^	0.04	−0.15	/	0.38^*^	0.24	/	0.42^*^
Rat 9	0.4^*^	0.12	0.44^*^	0.16	0.29	0.19	0.44^*^	/

Pearson correlation coefficients between the saliency maps obtained for Objects 1–4 and those obtained for a simulated ideal observer. (

* *p < 0.05, permutation test)*.

The motivation to compute group average saliency maps also for the animals tested with Stimulus Set 2 (in addition to the rats tested with Stimulus Set 1, as originally done in Alemi-Neissi et al., 2013), in spite of the large between-subject variability of the saliency patterns obtained with Object 3 and 4 (see Figure 11), was that, as previously discussed, the overall object regions (i.e., top or bottom part of the stimulus) containing mostly salient (or anti-salient) features were broadly preserved across rats (although the finer-grain features' patterns were only minimally preserved). Therefore, computing rat group average maps would still allow enhancing those features that were more consistently relied upon across subjects, by averaging out the idiosyncratic aspects of individual rat strategies. The resulting patterns of critical features extracted from the average saliency maps (see red and cyan patches in Figures 12A,B, top rows) indicate that, for most views, the salient features were generally located in the upper lobes of Object 3 and in the central lobe of Object 4 (or, occasionally, in Object 4's lower margins).

Saliency patterns that were broadly consistent with the ones obtained for the “average rat” were found for the ideal observer too (compare the bottom rows of Figures 12A,B to the top rows). For instance, in the case of Object 3, the salient region obtained for the ideal observer also covered most of the upper lobes, although not the tip of the right lobe (as found, instead, for the average rat). This salient region extended to the central part of the stimulus for all tested views (Figure 12A, bottom row), while this was the case only of 2 out of 8 views for the average rat (i.e., the default and the position right views; see Figure 12A, top row). Object 4 had a large salient region in the bottom part of the central lobe, which extended to the stimulus lower margins, giving rise to a U-shaped salient feature (see Figure 12B, bottom row). While this pattern was quite consistent with the overall saliency pattern observed for the average rat, in the case of the ideal observer (but not of the average rat) the tip of the upper-right lobe was also salient for most views.

For every object view, the extent to which average and ideal saliency maps matched was quantified by computing the Pearson correlation coefficient (reported under each pair of saliency maps in Figure 12). This coefficient was significantly higher than expected by chance for all views of Object 3, and in 4 out of 8 cases for Object 4 (*p* < 0.05; permutation test; see Materials and Methods and Alemi-Neissi et al., 2013 for details). This implies that, similarly to what found for Stimulus Set 1 (see Figure 10 in Alemi-Neissi et al., 2013), also for Stimulus Set 2 rat recognition strategy was, on average, consistent with an optimal strategy. That is, rats made, on average, close-to-optimal use of the discriminatory information afforded by Objects 3 and 4, in spite of their lower discriminability, as compared to Objects 1 and 2. This was definitely the case for Object 3 (for which the correlation was significant at all tested views). As for Object 4, the correlation with the ideal saliency map was either null, or failed to reach significance, in all those cases where the average map was highly scattered (i.e., see the default, size, azimuth right, and position right views shown in Figure 12B, top row).

As mentioned before, the saliency maps obtained for the ideal observer were also compared with the saliency maps obtained for individual rats. The result of these comparisons (i.e., Pearson correlation coefficients and their significance) are reported in Table 2, for all the rats belonging to the two experimental groups (rows) and all the views that have been tested for each rat (columns). The highest correlation values were observed for Object 2, which also yielded the largest fraction of significant correlations (~85%; 29/34 instances) along with Object 3 (~85%; 17/20 instances). By comparison, ~38 and ~35% of the correlations were significant, respectively, for Object 1 (13/34 instances) and Object 4 (7/20 instances). This indicates that, also at the level of individual rats, there was a good consistency with a strategy that makes close-to-optimal use of the objects' discriminatory information.

At first, having observed this agreement between rat (both average and individual) and ideal saliency maps, regardless of the similarity of the stimulus pair the animals had to discriminate (i.e., also for the objects belonging to Stimulus Set 2), could sound surprising. In fact, as previously shown in Figures 5, 11, the patterns of salient features obtained for Objects 3 and 4 were poorly reproducible across views and rats, and one could wonder, given such variability, how they could be significantly correlated with the saliency patterns of the ideal observer. However, it should be considered that the Pearson correlation coefficients reported in Figure 12 and Table 2 measure the similarity between patterns of saliency map values, each taken as a whole (i.e., the patterns of gray shades shown in Figures 4, 12), and not the precise overlap between those few individual saliency patches that crossed the threshold to be considered significantly salient (i.e., the red patches in Figures 4, 12). Therefore, the consistency between the saliency maps obtained for the rats and the ideal observer should be interpreted as a tendency, for rats, to exploit those relatively large object regions that are generally more informative about object identity. However, within these regions, whether the precise pattern of individual salient features (i.e., their location, size, shape, etc.) was also preserved across views and rats strongly depended on the structure and discriminability of the target objects (as shown in the previous sections).

As previously reported for the objects of Stimulus Set 1 in Alemi-Neissi et al. (2013), also in the case of Stimulus Set 2 the saliency map found for the view of a given object roughly resembled the negative image of the saliency map found for the matching view of the other object (see Figures 4, 12). Such a “phase opponency” (or “reversed polarity”) is especially noticeable in the case of the ideal observer (i.e., compare the bottom rows of Figures 12A,B), but is clearly observable also for the saliency maps of the average rat (i.e., compare the top rows of Figures 12A,B). To quantify this phenomenon, we computed the Pearson correlation coefficient between saliency maps of matching object views, for both the average rat and the ideal observer (see Table 3). The correlation coefficients ranged between −0.75 and −0.93 in the case of the ideal observer and they were all significantly lower than expected by chance (*p* < 0.05; permutation test). This suggests that the optimal extraction of the discriminatory information afforded by two objects naturally leads to saliency maps with reversed polarity across matching views of the two objects. Also in the case of the average rat, most correlation coefficients were significantly lower than expected by chance (*p* < 0.05; permutation test). Although, on average, their magnitude was lower than for the ideal observer (−0.8 ± 0.02 ideal vs. −0.4 ± 0.09 average rat; *p* < 0.05, two-tailed paired permutation test), this finding further confirms that rat recognition strategy was broadly consistent with the optimal extraction of discriminatory object information.

**Table 3:** **Phase opponency of the saliency maps obtained for matching views of Object 3 and 4**.

	**Default**	**Size**	**Azimuth left**	**Azimuth right**	**Position left**	**Position right**	**In-plane left**	**In-plane right**
Average rat	−0.27	0.11	−0.64^*^	−0.51^*^	−0.61^*^	−0.28^*^	−0.37^*^	−0.73^*^
Ideal observer	−0.84^*^	−0.75^*^	−0.8^*^	−0.81^*^	−0.92^*^	−0.93^*^	−0.79^*^	−0.82^*^

Pearson correlation coefficients between the saliency maps obtained for matching views of Object 3 and 4 (i.e., the same maps shown in Figure 10). For both the average rat (top row) and the ideal observer (bottom row), the significance of the correlation was assessed by a permutation test (

* *p < 0.05)*.

## Discussion

### Summary

The goal of this study was to investigate the influence of objects' structural complexity and similarity on rat recognition strategy. As a follow-up to one of our recent studies (Alemi-Neissi et al., 2013), we exploited the same classification image method used there, known as the Bubbles, which has been previously applied to human (Gosselin and Schyns, 2001; Nielsen et al., 2008), monkey (Nielsen et al., 2008), pigeon (Gibson et al., 2005) and, recently, rat vision studies (Vermaercke and Op de Beeck, 2012; Alemi-Neissi et al., 2013). This approach allowed the identification of the visual features that are critical, for rats, in order to correctly discriminate two objects, in spite of both affine (i.e., size/position changes and in-plane rotations) and non-affine (i.e., azimuth in-depth rotations) transformations. The comparison between our previous findings (Alemi-Neissi et al., 2013), obtained with structurally dissimilar objects (i.e., Stimulus Set 1; see Figure 1A, left panels) and our present findings (i.e., Stimulus Set 2; see Figure 1B, left panels) uncovered several key aspects of rat recognition strategy.

First, when required to discriminate objects with prominent, easily distinguishable structural parts (as in the case of Stimulus Set 1), rats were able to effectively process these parts and use them as markers of object identity (see Alemi-Neissi et al., 2013, for details). This resulted in a perceptual strategy where the diagnostic (salient) features closely matched the structural elements of the target objects (e.g., the central region of Object 1's top lobe or the tip of the lobes of Object 2; see Figure 6 in Alemi-Neissi et al., 2013). On the other hand, rats that faced a harder discrimination task (Stimulus Set 2) relied on smaller, more numerous and more scattered object features, often failing to display a clear match with the objects' structural parts (see Figures 4, 6–10 for a quantitative comparison among the two stimulus sets).

Second, for the rats tested with Stimulus Set 1, the recognition strategy was remarkably stable (i.e., view-invariant) in the face of variation in object appearance (see Figure 6 in Alemi-Neissi et al., 2013). This was shown by the large overlap found (for both Object 1 and 2) between the patterns of salient features of different views, after aligning one view back onto the other (i.e., see the aligned overlap axis in Figure 8B of Alemi-Neissi et al., 2013). The recognition strategy of the objects belonging to Stimulus Set 1 was also highly reproducible across rats (see Figure 11). On the other hand, rats tested with Stimulus Set 2 displayed a more variable pattern of diagnostic features across object views (see Figures 5B–D), and a higher inter-subject variability (see Figure 11), which are suggestive of a more view-dependent recognition strategy. Importantly though, for both groups of rats, no trivial, screen-centered strategy could explain rat recognition behavior (i.e., pairs of raw and aligned overlap values lay mostly below the diagonal not only in Figure 8B of Alemi-Neissi et al., 2013, but also in Figure 5B of the present study).

Third, rat recognition performance was, for both groups of rats, typically larger than chance over large extents of the tested transformation axes, with a substantial drop that was observed only for extreme transformation values, especially in the case of Stimulus Set 2 (see Figure 3).

### Interpretation, implications, and limitations of our findings

As mentioned in the Introduction, view-invariant theories (in their strongest version) posit that, across changes in object view, there should be no change in recognition performance—as long as the diagnostic features are accessible, the response of the system remains invariant. By comparison, view-dependent theories hypothesize that changes in the object appearance will generally result in variation of recognition performance, since objects are represented according to how they appeared when originally learned (for a review, see Tarr and Bülthoff, 1998; Lawson, 1999; Biederman, 2000). Since both groups of rats displayed a modulation of recognition performance, one could argue that rats, in general, rely on a recognition strategy that is mainly view-dependent, and becomes view-invariant as a result of training—as shown for monkeys and pigeons, when tested with unfamiliar, hard-to-discriminate objects; (Logothetis and Pauls, 1995; Wasserman et al., 1996; Spetch et al., 2001; Spetch and Friedman, 2003; Nielsen et al., 2006). However, even in “highly invariant” visual systems, like the human one, perfect invariance of the recognition performance is virtually never achieved (Biederman, 1987, 2000; Afraz and Cavanagh, 2008, 2009). More importantly, our classification image approach allowed going beyond what could simply be inferred based on performances, because it provided a direct assessment of rat perceptual strategy and its invariance. As reported in our previous study (Alemi-Neissi et al., 2013), the analysis of the patterns of diagnostic features showed, for Stimulus Set 1, a consistency in “tracking” the diagnostic features across all or most the object views the animal faced. From this, we can infer that rats are able to actively detect and extract discrete object features, which are relied upon regardless of the transformations the objects may undergo. The present study suggests that the crucial requirement for this ability to emerge is the distinctiveness of the objects, in terms of their structural similarity and the presence of “well affordable” object-specific features. Similarly to what has been reported for humans (Newell, 1998; Hayward and Williams, 2000; Spetch et al., 2001; Vuong and Tarr, 2006), rats can make use of a view-invariant strategy when confronting easily discriminable objects. Conversely, a view-dependent recognition strategy will emerge as the result of a discrimination involving visually (and structurally) similar objects. This appears to be the case of Stimulus Set 2, where the spread of salient features found for both Object 3 and 4 suggests that the rats recognized these stimuli using a novel set of features for each view.

Taking into account the larger stability of both the recognition performances and the patterns of diagnostic features observed for Stimulus Set 1, as compared to Stimulus Set 2, we can conclude that rat recognition strategy can be more or less view-invariant, depending on the structural similarity of the target objects. Objects that are structurally dissimilar are recognized by a lower number of diagnostic features, which map onto the objects' distinctive parts across a variety of transformation axes and magnitudes (view-invariant strategy). Objects that are structurally similar are recognized through a more variable, more scattered and more numerous set of features (implicating that learning at each tested view is needed; viewpoint-dependent strategy). But view-invariant and view-dependent strategies are not mutually exclusive. As observed for humans, “it is likely that the visual system employs them all to some degree to achieve object constancy” (Lawson, 1999). As for rats, this is in agreement with a recent report (Tafazoli et al., 2012), demonstrating how these animals can spontaneously (i.e., without any training) generalize their recognition to novel object views (view-invariant strategy), although the accuracy of the discrimination improves when training is provided (view-dependent strategy).

It is worth mentioning that, according to modern theories of object recognition, be they based on hierarchical feedforward processing (see, for example, Riesenhuber and Poggio, 1999) or recurrent, error-driven computations (see, for example, O'Reilly et al., 2013), the view-invariant vs. view-dependent debate may appear outdated (Hayward, 2003). However, being concerned with the role of learning and memory in object recognition, and their impact on object representations at the neural level, such a distinction still provides a rather useful theoretical framework to understand the invariance problem. For instance, the object recognition model proposed by Riesenhuber and Poggio (1999) explicitly embodies both view-invariant and view-dependent computations in the same feedforward architecture. At the first stages of processing, iterated AND-like and OR-like computations implement general-purpose banks of local feature detectors, which respond to subportions of visual objects with increasingly complex shape tuning and tolerance to size and position changes. Instead, the upper stage of the model (corresponding to monkey inferotemporal cortex) is made of “view-tuned” units, i.e., simulated neurons that selectively respond to different views of the objects that the model has been trained to discriminate. Other experimental and computational studies (DiCarlo et al., 2012; Wyatte et al., 2012; O'Reilly et al., 2013; Tang et al., 2014) have recently highlighted the importance, in object recognition, of coupling feedforward computations (based on little or no re-entrant processing) with recurrent computations (based on within-area, error-driven learning). Such a coupling could play a key role at the latest stages of processing, as well as under particularly challenging viewing conditions (e.g., when object appearance is occluded, degraded, or dramatically shifted from its “canonical” view, as in the case of masking or in-depth rotation). The combined findings of our current and previous studies (Zoccolan et al., 2009; Tafazoli et al., 2012; Alemi-Neissi et al., 2013; Zoccolan, 2015) fit within this theoretical and experimental framework, suggesting that rat invariant recognition is achieved by combining the automatic tolerance granted by local, partially invariant feature detectors with the fuller invariance provided by acquired, view-specific object representations.

Finally, our data show that, even in the case of structurally similar objects, the saliency maps underlying rat recognition strategy partially (but often significantly) overlap with those obtained for a simulated ideal observer engaged in the same invariant recognition task (see Figure 12). As discussed in the Results, this implies a tendency, for rats, to select the diagnostic object features within those relatively large object regions that are the most informative about object identity (although the across-view and across-rat reproducibility of the specific patterns of diagnostic features will strongly depend on the discriminability of the target objects).

It is important to point out that our current study rests on behavioral data collected from a rather small number of rats (3, i.e., half of the animals that were tested in our previous study, Alemi-Neissi et al., 2013), thus possibly limiting the generality of our conclusions. This would be the case, if our results were based on comparing group average performances (as in Figures 2A, 3). On the contrary, the conclusions of our study mainly rest on comparing the reproducibility of rat recognition strategy across subjects and object views. Since many different object views were tested and, for each view, multiple salient features were obtained, the most crucial data analyses reported in the study (shown in Figures 5–11) are based on tens of data points, thus allowing an adequate statistical sample and a robust assessment of rat recognition strategy.

Taken together, the results presented in this study suggest that, similarly to what observed for humans, also for rats, transformation-tolerant recognition can flexibly rely on either view-invariant representations of distinctive object features or view-specific object representations. Given the extraordinary potential of the rat as a model to dissect neuronal functions at the molecular, synaptic, and circuitry levels (Margrie et al., 2002; Ohki et al., 2005; Lee et al., 2006; Greenberg et al., 2008; Deisseroth, 2011; Fenno et al., 2011; Egger et al., 2012; Tye and Deisseroth, 2012; Meyer et al., 2013), our findings suggest that rat studies could significantly advance our understanding of the formation and maintenance of transformation-tolerant object representations in the visual cortex.

### Conflict of interest statement

The authors declare that the research was conducted in the absence of any commercial or financial relationships that could be construed as a potential conflict of interest.
